# Transfer-consultations for young people: how do we do?

**DOI:** 10.1186/1546-0096-9-S1-P272

**Published:** 2011-09-14

**Authors:** G Teilmann, K Boisen, J Pødenphant, M Zak, S Nielsen

## Background

The need of well-planned transition programmes for young people is well established, and there is growing evidence of the benefits in terms of increased health realted quality of life, better disease knowledge and patient satisfaction. However, we still need a better understanding of the optimal models and the outcomes of establishing such activities.

## Aim

To establish well-planned transfer-consultations for young people with rheumatic diseases to ensure youth-friendly uninterrupted health care provision and evaluate the outcomes.

## Methods

A mini-survey (N=10) and two individual interviews was conducted among young patients in the pediatric out-patient clinic to explore the young peoples wishes for a youth-friendly daily praxis as well as wishes concerning transfer to the adult department. Based on these experiences we introduced shared transfer-consultations in collaboration between rheumatologists from the pediatric and the adult department for adolescents with rheumatic diseases as a pilot-project. Participants in the transfer consultaions were the young patient as well as a rheumatologist from the pediatric (SN/MZ) and the adult department (JP). 30 adolescents have participated so far.

## Results

Feed-back from young patients and doctors during the pilot-phase has over-all been positive. The pilot phase has demonstrated a need for involving young people in the planning process. We are currently developing youth friendly information material about transfer from pediatric to adult rheumatolgy as well as a written policy for transfer. In addition a systematic evaluation of the transfer-consultations is needed. Results from semi-structured interviews with adolescents and doctors who have participated in a transfer-consultation will take place in May-August 2011 and will be presented at the conference.

## Conclusion

Young patients and doctors are positive towards shared transfer-consultations. Involving young people in the planning proces has shown to be fruitful. A written policy and a systematic evalutation is under preparation.

